# Insights Into Needs and Preferences for Mental Health Support on Social Media and Through Mobile Apps Among Black Male University Students: Exploratory Qualitative Study

**DOI:** 10.2196/38716

**Published:** 2022-08-31

**Authors:** Kofoworola D A Williams, Clarisa Wijaya, Caitlin A Stamatis, Gabriel Abbott, Emily G Lattie

**Affiliations:** 1 Department of Preventive Medicine Feinberg School of Medicine Northwestern University Chicago, IL United States; 2 Department of Psychiatry and Behavioral Sciences Feinberg School of Medicine Northwestern University Chicago, IL United States; 3 Weinberg College of Arts & Sciences Northwestern University Chicago, IL United States; 4 Department of Medical Social Sciences Feinberg School of Medicine Northwestern University Chicago, IL United States

**Keywords:** Black or African American men, college, mental health, social media, mobile apps, mobile phone

## Abstract

**Background:**

Black college-aged men are less likely than their peers to use formal, therapeutic in-person services for mental health concerns. As the use of mobile technologies and social media platforms is steadily increasing, it is important to conduct work that examines the future utility of digital tools and technologies to improve access to and uptake of mental health services for Black men and Black men in college.

**Objective:**

The aim of this study was to identify and understand college-attending Black men’s needs and preferences for using digital health technologies and social media for stress and mental health symptom management.

**Methods:**

Interviews were conducted with Black male students (N=11) from 2 racially diverse universities in the Midwestern United States. Participants were asked questions related to their current mental health needs and interest in using social media platforms and mobile-based apps for their mental health concerns. A thematic analysis was conducted.

**Results:**

Four themes emerged from the data: current stress relief strategies, technology-based support needs and preferences (subthemes: mobile-based support and social media–based support), resource information dissemination considerations (subthemes: information-learning expectations and preferences and information-sharing preferences and behaviors), and technology-based mental health support design considerations (subtheme: relatability and representation). Participants were interested in using social media and digital technologies for their mental health concerns and needs, for example, phone notifications and visual-based mental health advertisements that promote awareness. Relatability in the context of representation was emphasized as a key factor for participants interested in using digital mental health tools. Examples of methods for increasing relatability included having tools disseminated by minority-serving organizations and including components explicitly portraying Black men engaging in mental health support strategies. The men also discussed wanting to receive recommendations for stress relief that have been proven successful, particularly for Black men.

**Conclusions:**

The findings from this study provide insights into design and dissemination considerations for future work geared toward developing mental health messaging and digital interventions for young Black men.

## Introduction

### Background

Mental health has become an increasingly prevalent issue on college campuses. In 2019, the American College Health Association released a survey with data from 67,972 college students from 98 institutions, including public, private, and 2- and 4-year institutions, within the United States [1]. In this survey, 60% of the students reported “overwhelming anxiety,” and 40% had difficulty functioning because of their depressive symptoms [1]. The mental health needs of college students have only increased during the COVID-19 pandemic [2,3]. During the pandemic, anxiety among college students increased by a factor of almost 7 [4], and there have been significant increases in clinically reported depression and anxiety symptoms among young individuals [5]. These increasing trends and risks of poor mental health have put growing strain on current systems of intervention such as face-to-face campus counseling centers [6,7].

Research shows that minorities and, more specifically, Black students bear a larger psychological burden because of extraneous stressors [8,9]. Specifically, among Black male students, the risk of experiencing poor mental health is increasing, resulting from overexposure to social and economic disparities rooted in race and racism [10-12] and compounded by stressors associated with college life [13,14]. However, Black male students seek and use mental health services at lower rates compared with their peers [15] and have been less likely to seek face-to-face therapy or counseling [15-17] for mental health concerns. From the current mental health literature, we see that this underuse of services is often due to discomfort and lack of trust as well as other attitudinal factors influenced by racial discrimination [17], medical mistrust [18], and stigma [19]. Despite efforts to attenuate such factors and improve mental health outcomes among minority populations [20], existing mental health resources such as campus counseling centers and services are chronically underused by minority students [21-25].

The current burden on health care systems and the continued underuse of formal services among minority students necessitate creative intervention strategies in digital mental health that are practical and user-oriented [26]. It is imperative that we find other avenues to address the unique mental health challenges currently facing college-aged Black men and to improve uptake and access to mental health support services [27,28]. Digital mental health programs may be an optimal way to engage Black men as they can be informal in nature and widely accessible [29]. Digital health equity research demonstrates that digital tools and mental health programs are effective in decreasing mental health issues and increasing access to services among minority populations [30]. However, many digital tools and health programs lack relevance to Black men and do not attenuate the social and attitudinal factors affecting young Black men’s engagement, use, and uptake of digital mental health resources [31] or their barriers to help seeking [24,32,33]. An approach to attenuating these factors and improving relevancy is to focus on understanding Black men’s current mental health needs and examine their use of digital tools such as social media and mobile technologies.

Social media and mobile technology use has increased significantly in the last few years [34], especially among young adults with mental illnesses [35]. Among young adults aged 18 to 25 years, 96% own a smartphone [36], and an overwhelming majority of those aged 18 to 29 years (84%) consistently use social media platforms [29]. More than 65% of Black American adults use social media platforms on a daily basis [29]. Social media provides a potential platform to address the increasing mental health needs of students in higher education in a way that is user-friendly, accessible, and convenient [35]. Social media is increasingly used as a stress-coping or mood management tool [37], indicating its potential as a support structure to reduce the effects of stress, provide social support, and encourage well-being among users [38-40]. Despite the high use of social media platforms and their increasing popularity among Black young adults [34,41,42], there is little research assessing the acceptability and effectiveness of social media in mental health promotion and prevention among young Black men. This is a missed opportunity as social media tools may be especially effective for disseminating novel health resources [43,44] and have been the leading medium for communication and dissemination of information during the pandemic [45].

Digital mental health programs should aim to use tools that are relevant to Black men and are already being used by Black men and to understand the feasibility and acceptability of such tools in mental health promotion. Recent studies have found that most (85%) social media users expressed interest in using social media to access programs for well-being to cope with their mental health symptoms [46]. This body of evidence suggests a high potential for digital and social media–based interventions to begin to bridge the gaps in mental health care. Moreover, our lack of understanding of the factors that affect Black men’s uptake and engagement with current digital health interventions and tools is a critical barrier to expanding mental health services and achieving mental health equity. Digital mental health programs would also need to include culturally sensitive components, center the experiences of men, and use components that are created with Black men’s needs and interests in mind [47-49]. The relevance of such programs and the development of culturally appropriate components can be learned from Black men if researchers create opportunities for Black men to engage with them in the research and design processes [48,50].

### This Study

This qualitative study was exploratory and aimed to identify college-aged Black men’s needs and preferences for using social media and mobile-based technologies to manage their stress and mental health symptoms. The specific aims were 3-fold: (1) to determine Black male college students’ current mental health challenges and strategies for stress relief, (2) to identify ways in which social media and mobile-based technologies can support their mental health needs and stress relief strategies, and (3) to identify digital health app features and social media platform features that are suitable for Black male college students who wish to manage their symptoms and seek help via nontraditional methods. The results will point to considerations for designing and developing social media–based mental health interventions that are tailored to Black male college students’ needs.

## Methods

### Overview of IntelliCare for College Students

This study was designed as a supplement to a larger study [51] focused on the dissemination and implementation of the mental health app IntelliCare for College Students. This app was adapted for college students from the IntelliCare platform, a self-guided mental health app [52] providing users with interactive apps, cognitive skills, behavioral strategies, and knowledge focused on depression and anxiety. Recent studies have shown significant improvements in anxiety and depression outcomes among IntelliCare users from various settings [51-53]. IntelliCare for College Students was implemented on the campuses of 2 racially diverse Midwestern universities. Preliminary findings from the larger study showed that Black male students were neither enrolling nor downloading this app despite it being free and accessible. Thus, this study was designed to investigate the potential reasons for low enrollment, identify mental health needs, and determine preferences for technology-based mental health and wellness resources among Black male students.

### Recruitment

Participants were recruited from 2 universities. The first author (KDAW) sent direct emails and flyers to relevant student organizations serving the general students, organizations serving Black and diverse students (eg, Black student associations and Greek organizations), and counseling centers on these campuses. Word of mouth and web-based advertisements via social media were also used to recruit participants. Eligible participants could not have engaged with the app that is a part of the larger study and had to self-identify as Black or African American men, be between the ages of 18 and 35 years, be enrolled as undergraduate or graduate students at one of the participating universities, and have access to a computer or mobile device with a Wi-Fi connection. Emails and recruitment materials included information about the study and contact information for research staff whom students could contact if interested in participating. For students who responded and were interested, a link was provided in a follow-up email to gather their consent and conduct eligibility screening through REDCap (Research Electronic Data Capture; Vanderbilt University), a secure web-based survey instrument. After a student was screened as eligible for the study and completed a web-based consent form, an interview session was scheduled.

### Study Procedure

Sessions were conducted by KDAW on a university-secured Zoom (Zoom Video Communications) account, and participants were given the option to have their video on or off for the interview. Participants were interviewed for 30 to 60 minutes using a semistructured interview guide to prompt an open-ended discussion related to the study goals and objectives. The interview guide was developed by KDAW in consultation with the senior author (EGL) combined with feedback received from research team members. The interviews focused on understanding the men’s mental health needs and ways in which social media and mobile-based apps could support their stress management strategies. Discussions also centered on the perceived acceptability of using social media and mobile-based platforms for mental health support and concerns. Sample interview questions included “Are you interested in using social media/mobile apps for stress management or for self-care?” and “What would make you interested or disinterested in using social media or mobile apps for stress management for self-care?” Prompt questions were asked for clarification purposes and as an aid to help students reflect on their own lived experiences as Black male students. The interviews were audio-recorded. After the interviews, participants were compensated for their time with a US $20 Amazon gift card.

### Data Analysis

Qualitative data were transcribed verbatim from the audio recordings by a professional transcription service. The transcripts were analyzed by 2 coders, who were both research assistants with training in qualitative analysis and worked under the supervision of KDAW. The coders used an inductive thematic analysis approach aligned with the methods described by Braun and Clarke [54]. First, an initial reading and open coding of each transcript was conducted for the coders to become familiar with the transcripts and data. The initial codes were grouped according to the study aims. KDAW and the 2 coders then developed a detailed codebook, which included the code name, the definition of each code, and illustrative examples such as quotations. The 2 coders independently analyzed the data using a web-based research app (Dedoose; SocioCultural Research Consultants) and met to compare, critique, and refine the coding process and discuss and resolve any disagreements. Finally, the coders, along with KDAW, derived salient themes from the data related to the study aims.

### Ethical Considerations

Before the interviews, all participants were informed of the study and the study’s purpose through recruitment emails during the screening for eligibility process and at the start of the interview sessions. Consent was gathered from all participants before their interview session. Participants were informed that the data collected for these interviews would only be used for research purposes and manuscript preparation. Importantly, all study procedures were approved (STU00205589) by the appropriate Institutional Review Board offices at the relevant institutions in which the study was conducted and from which the participants were recruited.

## Results

### Sample

A total of 11 men (n=4, 36% undergraduate and n=7, 64% graduate students) consented and completed the interview sessions. Their ages ranged from 18 to 35 (mean 26.90, SD 7.11) years. Table 1 shows the sample characteristics.

**Table 1:** Sample characteristics (N=11).

University and grade level		Age (years)
**University 1**		
	Undergraduate	20
	Undergraduate	18
	Undergraduate	20
	Undergraduate	19
	Graduate	26
	Graduate	28
	Graduate	34
**University 2**		
	Graduate	25
	Graduate	35
	Graduate	35
	Graduate	35

### Themes

#### Overview

Four major themes emerged from the data: (1) current stress relief strategies, (2) technology-based support needs and preferences, (3) resource information dissemination considerations, and (4) technology-based mental health support design considerations. Table 2 presents a summary of the primary themes and subthemes, which are described in detail in the following sections.

**Table 2:** Brief summary of the themes.

Number	Primary theme	Subthemes
1	Current stress relief strategies	—^a^
2	Technology-based support needs and preferences	Mobile-based support Social media–based support
3	Resource information dissemination considerations	Information-learning expectations and preferences Information-sharing preferences and behaviors
4	Technology-based mental health support design considerations	Relatability and representation

^a^There was no subtheme for the primary theme listed.

#### Theme 1: Current Stress Relief Strategies

The men reflected on their established stress management activities. In general, participants cited the importance of creating dedicated time for self-care. A participant described this as “disconnecting from work, or school, and just taking time to take care of my mental health” as a strategy for dealing with their stress. In terms of specific activities for stress management, common ways of managing stress were either technology-enabled, such as watching YouTube videos, or nontechnology-enabled, such as going for a walk. Several men talked about how listening to music or watching funny videos on their computers or on television helped them relieve stress. For example, a participant described watching videos to boost his mood:

I believe part of the strategy to actually boost your mental wellness is to be happy from inside, to have this kind of joy. That’s why I kept on mentioning funny videos because when you laugh, of course, you know that it’s part of mental wellness as well. You laugh, you just forget about some worries and some stress that might be holding you down.

Another student went into detail about specific television shows that provide stress relief:

I mean, TV does that in general, but Insecure specifically just takes me out of my problems of the day and the stress of the day, and I can just focus in on what’s happening. Just how engaging it is.

Multiple men discussed how they would go for walks, work out, and go to the gym to relieve their stress and see friends. A participant strongly emphasized the social component of seeing friends as a means of stress relief:

For me, I normally go to friends. Even if you’re required to drive, I go and then have some nice times with them and feel good before coming back.

Multiple men mentioned Calm, a meditation app, as a way in which they were currently relieving their stress. The men used Calm to meditate and as a resource to get their mind off stressors, such as schoolwork, or right before engaging in something stressful, such as work responsibilities. Some students mentioned appreciating having the time to sit, relax, and be still:

[It] just allows me to recalibrate myself and not get so stressed out and focus on sitting and thinking really deeply about how you’re feeling and what you’re going through.

Some men discussed already engaging with social media to disconnect from their current environments or seek positive interactions. For example, a participant reported using social media platforms to look for motivational quotes and wanting to obtain encouragement from positive words on social media platforms.

#### Theme 2: Technology-Based Support Needs and Preferences

There was discussion about how mobile-based apps and social media could support the men’s current stress relief and management practices. The men were primarily interested in using social media and mobile-based apps for mental health support and stress management if social media and apps could connect them with resources, provide words of affirmation and encouragement, and provide stress relief recommendations.

##### Mobile App–Based Support

Students mentioned several areas in which mobile-based apps could support their mental health, including promoting physical activity or exercise, providing them with a space to watch television and movies (and offering suggestions), providing reminders to engage in activities that relieve stress, and helping them maintain a routine. Help with maintaining a routine, particularly through notifications, was a commonly discussed support preference. Regarding notifications, the men wanted to be reminded or encouraged to engage in stress relief activities or activities that allowed them to take care of themselves. A participant described how he could envision notifications being useful:

Maybe have the app say, “What do you wanna do to help you feel better? What do you think you can do to mitigate your problems?” and say, “Well, hey, I like to ride bikes.” So, maybe have it set to say, “Hey, it’s Saturday,” or whatever. “Don’t forget that you wanted to ride your bike,” or do whatever you wanna do. For me, it’s really I need an app that has a feature to remind me. I know you have life going on, but don’t forget about this that you put time and effort into thinking about. So, honestly, notifications to help keep you on track is kind of the best way for an app to get you back on track.

Another participant mentioned that notifications could be important and help with their goal setting:

Not only ask you how you’re doing—I think with a freeform answer, that would be hard, but preset things so that it could use that information to act upon it or give you some kind of notification, but also to set up a goals, like what do you wanna accomplish this week or this month. And let’s say somebody like me who’s busy but on the weekends.

The men also discussed wanting mobile apps to be synced with their calendar and notify them of time and space to take to mediate or debrief from their day. Students described wanting apps to notify them with inspirational quotes, videos, or something funny that encouraged them to do something positive and have positive thoughts. A participant was particularly interested in being sent messages about positivity and “stress relieving quotes.”

Another topic that emerged was how apps could provide some of the benefits of social media without the known detriments of existing social media tools to mental health. A student explained their views on how a mental health app could be a form of “healthy social media”:

I think an app can work as a piece for healthy social media because social media, in general, can being very draining. So, whether it’s just sharing ideas with people, or having a platform to where it’s kinda like, the resource for different activities, like mindfulness actives, and self-reflect activities, or actualization activities, or introspection. So, I think stuff like that, and just have little activities and a way to where you can communicate with people outside of just the craziness that’s going on. That way you can have a platform where you can like, ah, feel good today. Then I go to this, and I might just do a cool crossword puzzle or something, and then I feel better. You know?

##### Social Media–Based Support

The men were asked about the types of mental health support they would want or expect to see on social media. Participants discussed wanting to see visual examples of people dealing with stress as a means for them to learn to deal with their own stress. A man discussed a desire to see the following:

...other people’s experiences on how they manage their stress, examples, more tutorial on the different kinds of stress and how to manage them. If there’s daily tweets or daily comments one could register for that would help one to manage stress each day, I think that would go a long way.

Furthermore, several participants described how social media platforms could provide knowledge about symptoms and offer helpful strategies for dealing with symptoms while helping reduce stigma around mental health among Black men. A participant stated the following:

Honestly, I think just talking about it because even as you say symptoms, I really don’t know all of the symptoms. So, a platform actually talking about mental health within the Black community. And even—well, mental health within the Black male community is too because that could definitely be separated. I feel like that would be important to—just to start a discussion.

The latter sentiment speaks to mental health stigma and the common perceived notion that mental health is not discussed among Black men or within the Black community. A student went further to talk about the importance of addressing mental health stigma by increasing awareness:

A big part is awareness because a big part of social media is bringing awareness to a lot of the issues going on in today’s society. So, well, there’s like, “Hey, have you been struggling with mental health? Here’s who to contact.” Mental health is real; making sure like eliminating the stigma around it because a lot of people just don’t talk about mental health, which isn’t healthy.

As with mobile-based app support, the men reported wanting social media to provide positive affirmations as a support method. A participant mentioned how receiving positive affirmations helped them “feel better” and “feel more confident.” Positive affirmations were also mentioned to create a supportive environment in which people could be vulnerable with each other. The men also discussed ways to promote mental health on social media, including partnering with established communities, organizations, or influencers. Specifically, a participant mentioned an Instagram feature, Instagram Live, that would be useful for promoting mental health via established entities. The student suggested having someone who could relate to Black men or a person who was knowledgeable of mental health and its etiology use Instagram Live to talk about mental health issues:

I’ve seen big or maybe it doesn’t have to be a big psychologist or whatever but having someone talk about mental health. Everybody tunes in and have an honest conversation. And again, with my previous point where I mentioned not it just being a one-way street where you tell us all the statistics about stress within Black men, stress within Black women, stress within the Black

community

This student continued to talk about how this may help with increasing awareness and starting conversations about mental health. The men also thought it would be helpful to partner with and use already established outlets on social media, for example, tapping into Instagram influencers who may be already focused on mental health needs and promoting mental health in communities or among students.

#### Theme 3: Resource Information Dissemination Considerations

Another major theme related to how the men might expect or want to hear about mental health resources and how they personally might share information related to mental health support or resources with peers.

##### Information-Learning Expectations and Preferences

One of the ways in which the men would expect to hear about mental health resources included their interest in attending conferences and seminars organized by their institutions and that aimed to inform students such as them about mental health support or programs. For example, a student mentioned interest in learning about mental health resources via new student orientations or student orientations specifically related to them. Specifically, if they were a premedical or international student, then they would prefer to hear about such support via that specific orientation or organization. Similarly, other ways the men discussed of hearing about resources for mental health were through admissions offices. A student went further to suggest what these programs and offices could do to disseminate information about mental health resources:

Those are good places where if you had a little card deck or something like that or a flyer, because as people are registering or getting admitted into school, the stressors of life come into play there a lot, so I think those would be good places for outreach.

Other suggestions included the men wanting and expecting to hear about resources through the mental health offices or “self-care parts of campus” and from campus-oriented wellness teams. The men would also expect to learn about resources from organizations and email listservs specific to people of color and from programs related to “ethnicity or religion or gender.” A participant recalled a particular outlet through which they typically received campus information to explain how they might expect and would like to receive mental health information:

...newsletters from our BGSA [Black Graduate Student Association]; they send out emails probably once a week regarding how to cope with schoolwork, like outside of class, and just things relating to people of color.

Another student mentioned a preference toward receiving mental health information from “niche little mailing lists like the BGSA; I pay attention to those as opposed to the student-wide ones.”

##### Information-Sharing Preferences and Behaviors

Through the interviews, participants shared potential ways in which they would be interested in sharing information about the mental health resources they were using or knew of through word-of-mouth discussion (ie, in-person, casual conversations with their friends). Word of mouth involved students having “casual” conversations with their friends and mentioning the resources. If sharing information about resources by phone, students would text words such as “This is a great app. Everybody should go check it out” and send the associated mental health resource website or app link along with that SMS text message. Participants mentioned that they might feel compelled to share and suggest a useful resource with the intention of helping another friend who might be dealing with issues or “feeling down.” They imagined that, if they were talking with their friends and realized that their friends felt down, they could take it as an opportunity to share the resource:

And also, just reaching out to friends, seeing how they’re doing and if they feel that they need a resource, I feel like bringing this up with them and sharing with others the resources that I use could be helpful.

As an example of how one might share a resource with a friend, a participant indicated that they might say something to the effect of the following:

“Hey, I’ve been using this app that’s pretty cool. It manages stress.” So, I mean, a lot of people have been feeling stressed out lately, so I could recommend it to my friends just casually.

A few men mentioned that their willingness to share a resource depended on whether they themselves used it and were able to benefit from it. A participant stated that “there is a common saying that if you have used, and it has worked for you, then you’ll be ready to recommend it.” Another participant suggested that, if they used it and it worked, they’d be willing to share it through the following avenues:

...ads and also sharing [with] groups everywhere on Facebook, on Twitter, sharing those types of information; shar[ing] it with friends.

Although word of mouth was popular for sharing information, the men discussed preferences using social media to share information about mental health resources. Using platforms such as Instagram and Twitter was frequently mentioned. The men would “tweet about it.” Hypothetically, a student discussed how, if they were using a resource, they would post comments and image-based posts showing personal “experiences concerning [mental health]; that way people who see it in such similar conditions would be interested in it.” The men also talked about the popularity of social media in addressing social issues, explaining this notion as a reason they might expect to hear and share information pertaining to mental health resources on social media:

Instagram, Snapchat, more Instagram, just because I know a lot of my friends on Instagram post a lot about social issues, whether it’s mental health or other parts of our society that are important. But even Snapchat too, just because I know that’s a lot of platforms that people might use.

A participant explained that social media is useful for promotion and would expect the following:

...a lot of events and apps or anything like [mental health–related] is really through social media. Social media, everybody uses that to post whatever is going on.

A participant went further to offer the sentiment that “any type of media promotion is usually how people at school learn about anything that’s going on at this point.”

#### Theme 4: Technology-Based Mental Health Support Design Considerations

##### Overview

The final theme focused on the men’s visual- and text-based preferences regarding features for social media–based or mobile app–based mental health resources. A common preference was for visuals that were primarily image-based or had minimal wording:

Pictorial representation of the message you are passing across gets people’s attention [better] than the words...If I see a picture of someone laughing or something so fascinating, I might be tempted to just click on it.

The men also mentioned wanting to see a social media platform that included testimonials, voice notes, or podcasts portraying individuals who looked like them and who had dealt with mental health issues. In these images or testimonials, they also would want men to describe the strategies they had used to deal with stress. The men also cited the use of clear and concise language as another factor to consider when designing platforms or resources for mental health support. Students explained this preference in relation to Instagram captions. The men would like Instagram captions to be simple in nature and less wordy, making them more attractive and easier to digest. The men noted that simple language would also be useful in suggesting a resource or stress relief activity.

The men noted that they would be drawn to platforms that provided resources for broad mental health concerns rather than specific disorders. A participant described how, as not everyone would resonate with having a specific mental illness, being broader with mental health messaging should be a consideration:

Mental health as opposed to specific terms for mental disorders...might not relate to everyone: “Well, that doesn’t really pertain to me right now,” so it doesn’t grab my attention. But yeah, I think something that’s more open to the entire umbrella.

##### Relatability and Representation

Emerging from this theme were discussions of the men wanting advertisements of resources to be portrayed in a way that resonated with their lived experiences. Probing questions were asked to gain more insight. Some men were able to provide examples, suggesting that platforms should include components or images that “portray men who look like them and are using resources or an app/platform for their issues” or “relate to their everyday lives as students or iPhone/droid users.” The men also described a preference for seeing resources or advertisements that showed Black men and used specific wording:

[I’m] drawn to someone who looks like me. And the words “Black men”...so I feel like I would personally be drawn to that once I—if I saw it somewhere.

The men were interested in seeing platforms that used words and phrases that focused on mental health and the stigma surrounding mental health challenges. The men discussed the lack of existing resources focused on helping and supporting Black men’s needs as well as the dearth of resources focused on dismantling the stigma associated with Black men and mental health:

And I’m a Black man, and back home in my culture we consider stress as a form of weakness, and people who go through a lot of stress. So, if more effort can go into putting the word out that going through stress is a normal thing and there are ways one could manage it. At this age now, I want to just have an idea of what this stuff is all about, like managing stress.

Another participant discussed wanting resources to use specific words to address stigma and provide help without judgment:

And then, the “doesn’t judge” part I think is really important. A lot of people don’t reach out because they’re scared of what people might think, so making that clear that we’re not going to judge, we’re here to help. That is comforting.

For digital mental health resources to be used by Black men and to increase men’s engagement with such tools, the resources, programming, and prevention efforts need to include visual- or text-based components that are visually appealing to men’s lives and lived experiences. Importantly, mental health efforts for Black men’s needs should be relatable and include evidence-based components that have been successful in addressing Black men’s mental health needs.

## Discussion

### Principal Findings

#### Overview

This study examined Black male college students’ needs and preferences for using social media and mobile-based apps for their mental health symptoms, stress management, and self-care. This qualitative study stemmed from a larger study [51] aimed at examining the uptake of a mental health app on 2 university campuses. In line with previous research indicating that minority students underuse existing mental health resources [24,55], early data indicated that Black male students were not enrolling in the parent study or downloading the stress management app despite its wide availability. To help address this limitation of the parent study, our qualitative study tackled a critical topic in digital health equity research—specifically, understanding and identifying digital mental health support needs and preferences among Black male students [31].

Primary existing methods of stress reduction included engagement with physical activity (ie, working out), watching television or YouTube videos, and spending time with friends. In terms of support needs and preferences, major themes included a desire for stress relief recommendations and positive messages, the helpfulness of notification-based support, and support via social media. Regarding dissemination considerations, overall, we found that the underuse of mental health services by Black male students does not reflect a lack of interest, nor does it reflect their lack of interest in learning about or sharing resources. Design considerations highlighted a need for visual-based content delivery and greater representation in how resources are advertised.

#### Stress Relief

Our findings regarding physical activity as an existing means of stress relief align with established literature on stress relief and mental health coping among young Black men in college; for example, Goodwill et al [56] similarly found that Black male students relieved stress through exercise, sports, or hobbies. Extending previous literature, our interviews revealed that physical activity provided stress relief for Black men. Specifically, within this study, physical activity included taking walks as a key form of stress relief [56]. Various men discussed taking walks as a form of self-care, which is a sentiment not often highlighted in the current literature.

A form of self-care and stress relief was mentioned in relation to social support which, in mental health research, is a common social-ecological factor that protects against mental health risk [57]. The men discussed spending time with friends and engaging in activities that promoted social support as a form of self-care and relief that helped them cope with stress. This sentiment aligns with recent broader mental health research examining the need for social components in apps. In recent work, we see that social support and social connectedness via social networking sites are preferred, associated with lower levels of depression and anxiety [58], and associated with higher engagement with apps [59]. We also see that, in other areas broadly related to mental health research, app users are more motivated to use the app and change their behavior if there is a social support component [60,61]. For example, Gowarty et al [61] found that participants with serious mental illnesses would be more motivated to quit if a smoking cessation app included a social support component. Notably, there is ambiguity in the mental health literature regarding which types of social support serve as a buffer for mental health among Black men and which do not. For the men in our study, engagement with friends was the primary source of social support that relieved their stress, which aligns with certain studies [57,62,63] but contrasts with others that suggest that social support via significant others or romantic partners is a stronger protective factor [64,65]. The stronger emphasis on social support from friends than from romantic partners in our study may be due to our college-aged sample; however, more research is needed examining the preferred social support among Black male college students.

Another important insight from this study involved the ways in which Black male students were already harnessing technology to cope with stress, which still points to design considerations for future digital health interventions. Multiple men reported watching YouTube videos and using social media platforms to seek encouragement from motivational quotes. This finding aligns with existing knowledge about preferred social media platforms from publicly available reports. Entities such as the Pew Research Center indicate that YouTube is the most popular of the social media platforms [29,42], and our results suggest that this preference also holds true among Black men.

#### Support Needs and Preferences

The men provided valuable insights into factors that make mental health services more relevant and effective for them. A common theme was a desire for notifications and reminders. Persuasive system design suggests that the personalization of digital tools (eg, through notifications) can promote engagement [66], leading to greater symptom improvement [53]. In line with this, the men in our study suggested that personalized notifications would help them maintain a routine and that calendar integration would be especially effective in promoting the carving out of space to practice stress relief methods. At the same time, this type of personalization is likely useful for the broader population and not necessarily specific to Black men. Black male students also discussed ideas on how to support self-guided digital mental health tools. Although there is a rich literature on using human support (in the form of a coach or clinician) to increase engagement with digital mental health tools [67], participants in this study were not asked about the potential role of a human supporter, nor did they independently identify human support alongside a digital mental health tool as a valued strategy [67,68].

#### Dissemination Considerations

The results of this study offer insights into Black male students’ preferences and needs in relation to learning about mental health resources. A finding from this study is the potential role of minority-focused or Black-focused campus-based organizations in disseminating mental health information to increase access to care for college-aged Black men. The men consistently mentioned wanting to learn about mental health support from campus-based organizations, particularly those specifically geared toward students such as them and that matched to their race or gender. This finding is consistent with literature suggesting that people from underserved populations are more receptive to information or resources delivered by people of a similar background, which promotes confidence and trust [69]. Notably, the results present a connection between dissemination preferences and potential ways to increase men’s engagement with mental health support. Overall, the men in this study were interested in mental health services but, without evidence of the services being effective, they were less likely to engage. The men expanded on this further, stating how they would be more likely to share information about a mental health resource if it *works* and, importantly, if it *works* for Black men. This finding speaks to the role of evidence-based mental health services and peer dissemination practices in promoting engagement with mental health support among Black male students and further promoting receptivity and believability. This consideration would be important to incorporate, aiding in the likelihood of mitigating factors related to stigma and medical mistrust [18,19] and improving young Black men’s access to and uptake of digital mental health support [27,28] down the road.

Previous research also underscores the importance of social media in disseminating political information for Black Americans [42] as well as for mental health promotion. The men discussed how they might be more open to specific social media platforms that promote mental health specific to college-aged Black men who are dealing with the same issues and concerns. Watkins et al [70] tested a Facebook-based intervention with content related to mental health education and support specifically for Black men. Qualitative analysis indicated that the men who engaged with this intervention had increased awareness not only of their own mental health and needs but also of the mental health needs of other Black men [70]. Our results similarly suggest that social media can be an acceptable tool for health promotion. The men in our study also discussed that they would willingly use social media to share mental health–related information with friends and peers, stating that social media would be an effective way of reaching them and their friends. The men indicated that they were already accustomed to receiving information about other health and social issues via social media, as were their friends. However, it is important to note that social media comes with limitations, namely, potentially misleading and inaccurate mental health resources and information, lack of mental health literacy, and the triggering of negative behavior [71]. Given the prevalence of social media use as a part of informal help seeking, future investigations should examine how to consider the quality and accuracy of mental health information and resources on social media and other web-based platforms for this population.

Nonetheless, future work should examine how best to promote mental health via social media outlets such as Instagram, Snapchat, YouTube, and Twitter. Previous qualitative research suggests that the dissemination of health information via social media can be achieved using various strategies, including contests, interactive campaigns (eg, with educational polls), and community building [72]. At the same time, it will be important for future studies to address the challenge of using social media as a platform for health promotion while reducing the risk of misinformation.

#### Design Considerations

##### Overview

A key finding regarding design considerations involved the role of social modeling in health promotion. Behavior change research suggests that the modeling of a behavior leads to optimal behavior change outcomes [73,74]. In this study, Black male students indicated that they would be more likely to engage with a resource on a mobile-based app or social media platform if it offered testimonials portraying positive mental health. This sentiment, indeed, speaks to the need for relatable and representative content in mental health app research.

##### Relatability and Representation

Although it was difficult for the men to explain what they meant by relatability, they primarily related this concept to representation. The men wanted to see themselves, see advertisements and resources made for them and that include images or message components related to their lives—as students, as Black men, and as emerging adults in college. The men discussed that seeing themselves in advertisements or components would make the resources more relatable. This design consideration aligns with the need for targeted design efforts in mental health research, which can diverge from research focused on inclusive design among other minority populations [75]. In our study, this preference for representative components and relatable content aligns more with targeted design in which these men prefer visual representations that are made for them and reflect Black men’s mental health needs. Importantly, the men also discussed that this preference stems from the observations that they do not see their lives, experiences, or needs reflected in current available resources and prevention efforts; therefore, their desire and comfort level to engage is limited. There is little research targeted to men from Black populations [76], which perpetuates disparities that further increase the risk of poor mental health and low treatment use among Black male students [77]. This gap also makes it difficult for researchers to develop mental health support that is effective in drawing Black male students in and engaging them in prevention efforts.

With targeted and tailored design approaches, specifically those that are culturally sensitive and appropriate, Black male students would be more likely to engage and resources would be better able to meet their needs [27]. This study focuses specifically on Black male students’ needs, creating a space for them to share those desires and needs, which will provide insights for designing targeted efforts that are visual, text-based, and reflective of the mental health experiences of Black men generally and in college settings. Thus, it will be important for campus-based practitioners and researchers to examine ways to tailor advertisements and intervention components specifically to Black men’s lived experiences. The current lack of representation in mental health research may prevent researchers and clinicians from accurately recognizing mental health difficulties at initial onset among Black men and understanding how mental health difficulties may present within the context of a college campus [78].

##### Stigma

This study provides insight into appropriate visual- and text-based components to include in design efforts and more representative digital tools focused on addressing stigma, a factor that remains a significant barrier to treatment use among minority populations [19,76,79]. In particular, in a study conducted by Lipson et al [76] with a sample size of 40,000 students (13,000 students of color), male students had higher levels of perceived stigma. In our study, Black male students discussed their desire for mental health apps and social media mental health support that included visual- and text-based components that indicated a need to normalize the discussion of mental health in the Black male community. The men discussed how, today, mental health remains a public health concern that is not discussed and almost ignored, a finding that aligns with previous research on mental health as a “taboo” subject among this population [80,81]. Social media is widely accessible, reaching individuals who are typically underserved and providing an outlet for these individuals to engage with mental health promotion materials and resources to increase mental health awareness [29]. Given the stigma surrounding traditional avenues of mental health discussion and treatment in the Black male community, social media–based mental health support may represent an initial, more accessible form of treatment to reach college-aged Black men as well as a means of normalizing conversations around mental health [80,81].

##### Audio and Visual Components

The findings also offer insights into specific preferences for mental health support on social media. The men reported a preference for audio and visual content delivery, such as experiencing mental health support through podcasts and listening to voice notes. There is little research focused on the role of podcasts and voice notes as methods for mental health promotion among Black men and, specifically, among Black men in college; however, there has been work focused on the role of podcasts in mental health education [82]. Willis [82] showed how podcasts create an opportunity for psychiatry residents not only to better understand course material related to mental health promotion but also to provide them with skills that can better prepare them to treat patients and aid in attenuating risk factors that influence mental health disparities. A similar approach may be effective in providing mental health psychoeducation for the purposes of increasing awareness and promoting mental health among Black male college students. Further work should be conducted to determine the role of podcasts in mental health promotion among Black male students and explore the feasibility of podcasting or audio in intervention work as a means of promoting desired behavioral health outcomes.

Our findings regarding design considerations suggest that text-based images are preferred avenues for health promotion. The men in our study preferred mental health support and resources that were image-based or had pictorial representations of mental health issues and safety. This is consistent with literature that suggests that methodologies related to photo elicitation are useful in promoting mental well-being [83,84] and should be further considered when designing mental health programs and interventions.

### Strengths, Limitations, and Future Directions

The strengths and limitations of this study point to directions for future research. Our study was exploratory and targeted a small Midwestern sample, potentially limiting the transferability of the findings to college-aged Black men from this region. Importantly, in relation to the study aim, there is research to suggest that saturation can be met with small sample sizes in qualitative research [85]. We do not know how focal characteristics from this study, such as digital mental health treatment preferences or openness to social media use for mental health treatment, vary across subgroups (eg, in rural vs urban areas and across regions). In addition, our sample included a range of ages and more graduate than undergraduate students, including exclusively graduate students from university 2. However, there was no qualitative difference in how needs varied between undergraduate and graduate students or between younger and older men. In future work, it would be worthwhile to examine the potential differences in how young Black men’s needs and preferences vary at different ages.

Selection bias may be a concern as well. The men in this sample may have been more motivated to participate in the study, especially if they heard about the study through a friend. However, we consider this a strength as well. These men’s thoughts and experiences are important within the mental health space and will provide unique insight into appropriate dissemination strategies and design ideas that can also be adapted for recruitment efforts that are better able to engage Black male students in future research [86,87]. The study findings should also be interpreted in consideration of the COVID-19 situation at the time. Given that the study was conducted during the peak of the COVID-19 pandemic, when college students were overwhelmed by a sudden transition to digital and computer-enabled classes and communications, the findings may potentially be influenced by their digital fatigue [51]. Participants’ perception of using web-based resources and digital tools may also be altered because of this.

### Conclusions

Our qualitative investigation to understand Black male students’ needs and preferences surrounding nontraditional methods of mental health promotion provides key insights into addressing current mental health access barriers. Our findings provide evidence for leveraging social media and digital tools to introduce mental health services in an informal way, making tools more widely accessible and more appropriate to meet their needs [88]. Digital tools and social media platforms are already being used to discuss mental health concerns [89] and have the potential to reach large numbers of individuals who are less likely to use traditional mental health services [29,42,90], such as Black men. As the pandemic continues to disrupt the ways in which mental health support is provided and increase the demand for services, it is critical to understand how minoritized groups are seeking mental health support and address key factors affecting adequate mental health care and overall access. The insights from this study can serve as a stepping-stone for researchers to develop work that is contextually and culturally relevant to Black male students, ultimately improving access and use rates of mental health services among Black men throughout their life span. Researchers, campus-based practitioners, and policy makers should aim to conduct work that promotes the role of social media and mobile-based technologies in mental health promotion and in supporting the unique mental health needs of Black male students and their communities.

## Abbreviations

REDCap: Research Electronic Data Capture
